# A Systems Biology-Based Investigation into the Pharmacological Mechanisms of Sheng-ma-bie-jia-tang Acting on Systemic Lupus Erythematosus by Multi-Level Data Integration

**DOI:** 10.1038/srep16401

**Published:** 2015-11-12

**Authors:** Lin Huang, Qi Lv, Fenfen Liu, Tieliu Shi, Chengping Wen

**Affiliations:** 1 TCM Clinical Basis Institute, Zhejiang Chinese Medicine University, 548 Binwen Road, Hangzhou, Zhejiang, 310000, China; 2 Center for Bioinformatics and Computational Biology, and the Institute of Biomedical Sciences, School of Life Science, East China Normal University, 500 Dongchuan Road, Shanghai, 200241, China; 3 School of Finance and Statistics, East China Normal University, 500 Dongchuan Road, Shanghai, 200241, China; 4 Biological Targeting Diagnosis and Therapy Research Center, Guangxi Medical University, Nanning, Guangxi, China

## Abstract

Sheng-ma-bie-jia-tang (SMBJT) is a Traditional Chinese Medicine (TCM) formula that is widely used for the treatment of Systemic Lupus Erythematosus (SLE) in China. However, molecular mechanism behind this formula remains unknown. Here, we systematically analyzed targets of the ingredients in SMBJT to evaluate its potential molecular mechanism. First, we collected 1,267 targets from our previously published database, the Traditional Chinese Medicine Integrated Database (TCMID). Next, we conducted gene ontology and pathway enrichment analyses for these targets and determined that they were enriched in metabolism (amino acids, fatty acids, etc.) and signaling pathways (chemokines, Toll-like receptors, adipocytokines, etc.). 96 targets, which are known SLE disease proteins, were identified as essential targets and the rest 1,171 targets were defined as common targets of this formula. The essential targets directly interacted with SLE disease proteins. Besides, some common targets also had essential connections to both key targets and SLE disease proteins in enriched signaling pathway, e.g. toll-like receptor signaling pathway. We also found distinct function of essential and common targets in immune system processes. This multi-level approach to deciphering the underlying mechanism of SMBJT treatment of SLE details a new perspective that will further our understanding of TCM formulas.

Traditional Chinese Medicine (TCM) is an ancient practice based on extensive knowledge and experiences accumulated over several thousand years and is both efficient and safe for the treatment of chronic diseases^1^. More than 100,000 TCM formulas take effect by concocting different natural products which are essential for therapy^2^. A formula is composed of herbs; thus, some studies have focused on the herbal extracts^3^ and their biological functions^4^. TCM formulas typically utilize multi-component therapeutics. These combinatorial treatments tend to increase therapeutic effects through the synergism of two or more herbs and decrease side effects through antagonism. The application of TCM in clinical practice has become increasingly important and has attracted attention as a source for drug development, such as the widely used drug, total glucosides of paeonia extracted from the root of *Paeonia lactiflora* that affects anti-inflammation, analgesia and immuno-regulation^5^. However, further investigation is still required to define the mechanism how herbs comprise any given formula function together^6^. Different herbal combinations have distinct treatment effects on individuals and contribute to the complexity of the mechanisms of TCM. As described in the “multiple components and multiple targets” concept of TCM, a formula exerts its therapeutic effect through interactions among the complex compounds of the medical herbs and the complex system of the diseased organism, which makes it extremely difficult for us to understand how these formulas treat diseases.

Although a great deal of effort has gone into unveiling the mechanisms behind TCM formulas, most remain unknown^7^. Thus, a systematic analysis of the complex mechanism behind TCM formulas is required. The rapid development of computational analyses and systems modeling approaches provide rich and substantial content of “biological networks”, and generate a new view of the life sciences and medical research^8^. Recently, the robustness of multiple systems biology platforms ensures the discovery of underlying molecular mechanisms and connections between drugs and their targets (e.g. proteomics studies on activated blood circulation of Chinese medicinal herbs)^9^. Newly developed algorithms and network-based computational models can integrate multi-level omics data and can optimize combinational regimens of drug development. The development of these tools encourages us to study medicinal herbs in view of network-based multi-target drug development^10^. Due to its robustness, sensitivity and adaptability, network-based drug combination discovery has the potential to provide a better understanding of the effects of Chinese herbal formulas, such as the neuroprotective mechanism behind Sheng-yu-tang^11^. Resources are available for these systematic analyses. For example, we previously published Traditional Chinese Medicine Integrated Database (TCMID, http://www.megabionet.org/tcmid/)^12^. TCMID is the largest integrated TCM database and provides the information of formulas, herbs, herbal ingredients, disease-related genes or proteins, diseases and drugs from large scale manually text mining and databases^13^. To be specific, we totally collected 46,914 formulas and 8,159 herbs by manual text mining, and data integration from various databases, including TCM-ID database (Traditional Chinese Medicine Information Database, http://tcm.cz3.nus.edu.sg/group/tcm-id)^14^ and *Encyclopedia of Traditional Chinese Medicines*^12^. The ingredients of herbs were derived from text mining, TCM-ID database, Herb Ingredients’ Targets database (HIT, http://lifecenter.sgst.cn/hit/)^15^ and TCM@Taiwan database (http://tcm.cmu.edu.tw/)^16^. The targets of ingredients were identified with literature evidences, HIT, STITCH (http://stitch.embl.de/)^17^, Online Mendelian Inheritance in Man (OMIM, http://omim.org)^18^ and DrugBank (http://www.drugbank.ca)^19^. Besides the comprehensive information, the database also contains online tools to present the relationships between herbs, ingredients and target genes in view of visual networks.

Systemic lupus erythematosus (SLE), a serious disease with no effective cure, is a multi-system autoimmune disease characterized by accumulation of anti-nuclear autoantibodies and various immunological abnormalities and is accompanied by excessive inflammatory responses in a wide range of organs^20^. This disease can be treated with immunosuppressant drugs, including cyclophosphamide, corticosteroids and other immunosuppressants^21^. However, the exact cause of SLE is not completely understood. Omics data has the potential to uncover the mechanism of SLE. For example, analysis of patient transcriptional profiles offered a means to investigate mechanisms relevant to human diseases on a genome-wide scale^22^. Indeed, by using genome-wide expression profiles, Damien Chaussabel and his colleagues identified transcriptional modules based on genes co-expressed in multiple disease datasets. They found 11 modules that contained differentially expressed genes related to SLE. A total of 9 out of the 11 modules were directly connected with the immune system (e.g., M1.1 included genes encoding immunoglobulin chains and the plasma cell marker)^22^. In TCM studies, Sheng-ma-bie-jia-tang (SMBJT), a popular TCM formula developed by Chinese medical sage Zhang Zhongjing, is widely used for the effective treatment of SLE^23^,^24^. It comprises six herbs with suggestive dose: Cimicifuga foetida (SHENG MA, 6 g), Carapax trionycis (BIE JIA, 3 g), Pericarpium zanthoxyli (SHU JIAO, 3 g), Realgar (XIONG HUANG, 0.5 g), Glycyrrhiza uralensis (GAN CAO, 6 g) and Angelica sinensis (DANG GUI, 3 g)^25^. The dose should be slightly modified according to the specific physical condition of each patient. To date, no significant side effects of SMBJT have been reported^23^. Although several studies focused on the experiments of SMBJT^24^, the pharmacological mechanism of SMBJT has not yet been fully elucidated.

To explore the potential pharmaceutical mechanism of SMBJT for the treatment of SLE, we analyzed its herbal targets obtained from the TCMID database. First, we discovered that the targets of SMBJT were significantly enriched in SLE related biological processes, e.g. regulation, signal transduction and metabolism. Considering whether targets were previously defined as SLE proteins, we classified targets into essential or common ones. Next, using a protein-protein interaction network we investigated the potential mechanism of essential and common targets. Finally, we adopted a functional module concept and surveyed module enrichment of SMBJT targets. Overall, this study proposed a systemic method to investigate the molecular mechanisms of SMBJT, which improves the understanding of this TCM formula.

## Results

### The herbs, ingredients and targets of the SMBJT formula

Six herbs are included in the SMBJT formula: Cimicifuga foetida, Carapax trionycis, Pericarpium zanthoxyli, Realgar, Glycyrrhiza uralensis and Angelica sinensis. Traditionally, Cimicifuga foetida has been used as a cooling and detoxifying remedy herb^26^ while Carapax trionycis can relieve fever^27^. Due to the significant functions in these effects, Cimicifuga foetida and Carapax trionycis are thought as the monarch herbs. At the same time, Pericarpium zanthoxyli, Realgar and Glycyrrhiza uralensis all assist in the clearance of heat and toxic materials. In addition to the main effects, Pericarpium zanthoxyli also relieves pain, while Angelica sinensis promotes blood circulation to remove blood stasis.

**Figure 1:**
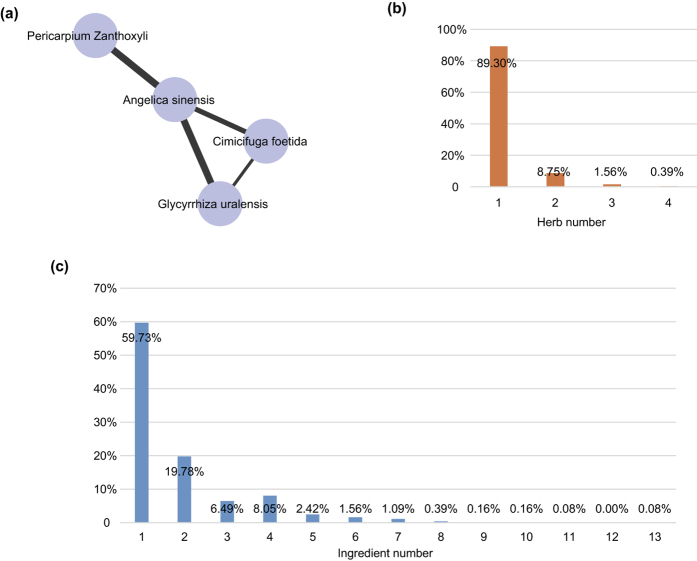
The relationships between herbs, ingredients and targets. (**a**) The relationships between the 4 herbs sharing the same ingredients. The links between herbs were constructed with the same ingredients in Supplementary Table S2. The width of the edge means the similarity between herbs in ingredients. (**b**) The component of targets correlated to different number of herbs. The numbers of herbs for targets were from 1 to 4, and each percentage was presented in the pie chart. (**c**) The component of targets correlated to different number of ingredients. The numbers of ingredients for targets were from 1 to 13, and each percentage was presented in the pie chart.

We obtained a total of 390 different ingredients of the six herbs from TCMID (Supplementary Table S1). Specifically, Cimicifuga foetida contained 42 ingredients, including bicuculline, visnagin, caffeicacid, etc. Carapaxtrionycis contains 3 ingredients, including vitamin D, collagen and keratin. Furthermore, there were 172 ingredients in Glycyrrhiza uralensis, 163 in Angelica sinensis, 16 in Pericarpium zanthoxyli and 3 in Realgar. Among the 390 different ingredients, we identified 8 ingredients that were present in more than one herb (Supplementary Table S2). These ingredients were obviously correlated with SLE. An ingredient (glycyrrhizic) inhibited the immunocomplex formation of 60S acidic ribosomal P proteins with their specific antibodies in sera from patients with SLE^28^, and another ingredient (limonene) had significant anti-inflammatory effects in murine dermal inflammation and wound-healing^29^. Besides, these ingredients that were shared between herbs might be the key ingredients in the biological function of the herbs. We further identified herb pairs sharing the same ingredients (Fig. 1a): Glycyrrhiza uralensis and Angelica sinensis (3 ingredients); Cimicifuga foetida and Glycyrrhiza uralensis (1 ingredient); Cimicifuga foetida and Angelica sinensis (2 ingredients); and Angelica sinensis and Pericarpium zanthoxyli (3 ingredients). Compared with single target therapies, these TCM ingredients shared by different herbs systematically promoted different biological responses (cooling, detoxifying, blood circulation and pain relief) in human body, which could be more efficient in complex disease treatment.

In TCMID, half of the 8 ingredients shared between herbs had related targets (Supplementary Table S2), and we totally obtained 1,267 highly confident targets of 93 ingredients. 89.3% of the targets were correlated with one herb, indicating the herb specific property of targets (Fig. 1b). However, 40.27% of the targets (Fig. 1c) were correlated with more than one ingredient, suggesting the “multiple components and multiple targets” in SMBJT formula. For example, interleukin (IL)-6 correlated with 13 different ingredients, e.g. glycyrrhizin and retinol (Supplementary Table S3). IL-6 is cytokine with the anti-inflammation and anti-allergy effects that were correlated with arthritis manifestation of SLE^30^. Another 4 proteins (IL-8, prostaglandin endoperoxide synthase 2 [PTGS2], tumor necrosis factor [TNF] and vascular endothelial growth factor A [VEGFA]) were correlated by at least 10 ingredients (Supplementary Table S3). The targets could be the most distinct factors for the formula, thus they were comprehensively investigated in the following analyses to uncover the molecular mechanism.

### Functional analysis of the 1,267 targets

We performed GO enrichment analysis of the 1,267 targets of SMBJT and found that they were significantly enriched in biological processes of signal transduction, biological regulation and metabolic processes (Supplementary Table S4). Formula targets were significantly enriched in top-ranked biological processes, such as signal transduction (29.1%), cell surface receptor-linked signal transduction (24.4%) and intracellular signaling cascade (16.41%). Additionally, the top enriched biological processes included many regulation-related processes, such as positive regulation, negative regulation, regulation of cell communication, regulation of cell proliferation and regulation of programmed cell death. In addition to signal transduction and biological regulation, the targets also participated in metabolic biological processes (e.g. oxoacid and cellular lipid metabolic processes).

The signaling and metabolic pathways were then investigated in detail. The targets were enriched in 40 pathways with p-values <0.05. The top 20 pathways (Table 1) belonged to three categories: organismal systems, metabolism, and environmental information processing and cellular processes. The identification of these pathways helped us to understand the functional contributions of the targets to SLE treatment. For example, two immune system pathways (chemokine signaling pathway and Toll-like receptor [TLR] signaling pathway) and two endocrine system pathways (adipocytokine signaling pathway and peroxisome proliferator-activated receptor [PPAR] signaling pathway) were implicated. Chemokines were small chemoattractant peptides about cell trafficking and thus were vital for protective host response in effective inflammatory immune response^31^. According to the KEGG database, the chemokine signaling pathway together with the adipocytokine signaling pathway were associated with a disease called Nuclear Factor kappaB Essential Modulator (NEMO) syndrome (incontinentia pigmenti). Interestingly, NEMO syndrome (an acronym of a mutated, non-functioning NF-kappaB gene) was associated with SLE^32^, and NF-kappaB was an essential modulator in the pathogenesis of SLE in the context of the complex immune deficiencies increasingly^32^. On the other hand, TLR family was responsible for sensing microbial pathogens and occupied an important position in innate immune responses^33^. TLR signals in B cells amplified anti-dsDNA autoantibody and enhanced one SLE characteristic, autoantibody production^34^. PPAR gamma expression, one of PPARs subtypes, was increased in SLE patients and regulated the inflammatory signal^35^.

Metabolism accounted for more than half of the top 20 pathways. These pathways belonged to five different categories (xenobiotics biodegradation and metabolism, metabolism of cofactors and vitamins, fatty acid metabolism, carbohydrate metabolism and amino acid metabolism) and were perturbed in SLE patients with literatures evidences. Metabolic disorders were detected in both human patients and mice with SLE, including disorders in amino acid metabolism in the SLE mouse model and significantly decreased tryptophan in human SLE patients^36^,^37^. In SLE patients, two important metabolites (citrate and pyruvate) were also decreased^38^, while essential fatty acid metabolism altered^39^.

### Overlaps between targets and SLE disease genes

**Figure 2:**
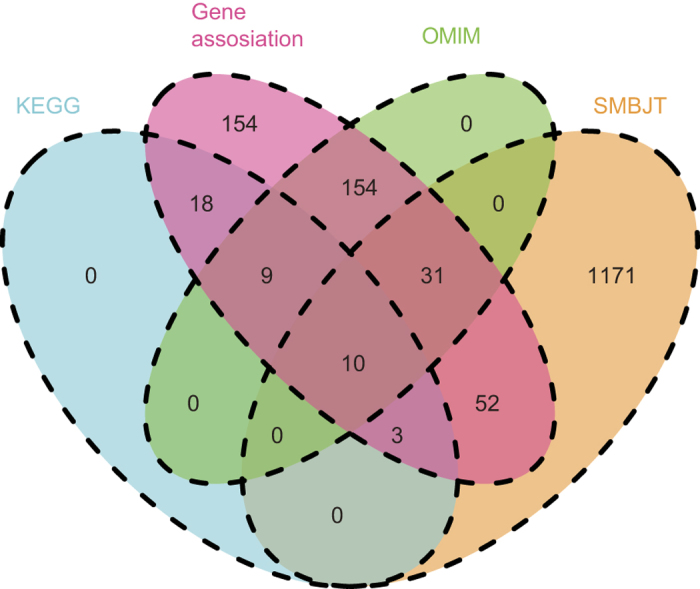
Comparisons between SMBJT targets and SLE disease proteins from different databases. The data in Gene association database are the most complete among the three databases. 96 targets in SMBJT are known SLE disease proteins. The remaining targets could be explained by the PPIs with SLE disease proteins.

We collected SLE disease genes from three databases (204 from OMIM, 431 from GAD and 40 from KEGG), and obtained a total of 431 SLE disease genes. We found that 96 targets of SMBJT were also defined as SLE disease genes in these databases (Fig. 2) and considered as essential targets. These targets included TLRs, ILs, chemokines, cytochrome P450 family, glutamate receptor, and adiponectin. These essential targets had the potential to significantly affect SLE with different mechanisms. For example, apolipoprotein E (APOE) was involved in the stimulus responses leading to changes in the state or activity of a cell or organism (in terms of movement, secretion, enzyme production, gene expression, etc.) as a result of a stimulus^40^. The polymorphisms of another essential target ATP-binding cassette, sub-family B (MDR/TAP), member 1 (ABCB1) could interfere with the clinical features of SLE^41^. The increased expression of one essential target (calcium/calmodulin-dependent protein kinase IV, CAMK4) was necessary for Th17 cell differentiation that was correlated to tissue inflammation in several autoimmune diseases, including SLE^42^. Three essential targets (vitamin D receptor [VDR], TLRs and APOE) played important roles in SLE pathogenesis^43^. Moreover, signal transducer and activator of transcription 1 (STAT1) were potentially useful indicators about the initiation, progression and maintenance of inflammation in SLE patients^44^. Beside essential targets, the remaining 1,171 targets (e.g. myeloid differentiation primary response 88[MYD88D], SRC proto-oncogene, non-receptor tyrosine kinase [p60-src], and macrophage migration inhibitory factor-like protein [MIF2]) were defined as common targets.

### Direct interactions between essential targets and SLE disease proteins

**Figure 3:**
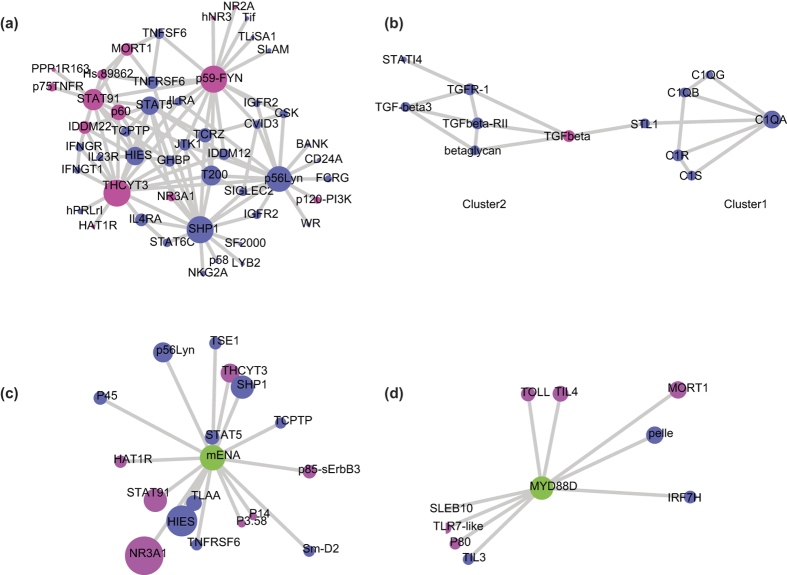
PPI sub-networks of SLE disease genes, essential targets and common targets. (**a**) A highly connected cluster in the PPI network of SLE disease genes and essential targets. (**b**) Two disease protein clusters connected by TGFbeta. (**c**) Sub-network of high-degree common target, mENA. (**d**) Sub-network of the high-degree, common target MYD88D. Blue, the products of SLE disease genes; red, essential targets; green, common targets.

To uncover the underlying mechanism of the targets, we applied PPIs to explore the functional relationships between targets and SLE disease proteins. A total of 910 targets and 329 disease proteins had PPIs. Among them, 94.7% of the targets either indirectly or directly interacted with 98.5% of the SLE disease proteins. The direct interactions between essential targets and disease proteins were presented in the network (Supplementary Figure S1). The network contained a highly connected PPI cluster, as well as PPI branches originated from the highly connected PPI cluster. The highly connected PPI cluster (Fig. 3a) included 49 nodes (35 SLE disease proteins and 14 essential targets). In this highly connected PPI cluster, 5 proteins (two SLE disease proteins: protein tyrosine phosphatase, non-receptor type 6 [PTPN6] and LYN proto-oncogene, Src family tyrosine kinase [LYN]; and three essential targets: STAT1, Janus kinase 2 [JAK2] and FYN proto-oncogene, Src family tyrosine kinase [FYN]) appeared to act as hub proteins based on their high degrees of association. These 5 proteins were closely correlated with SLE. For example, PTPN6, STAT1 and JAK2 were involved in JAK-STAT1 signaling pathway. Experiments confirmed the detection of hyperactivation of the JAK-STAT1 signaling pathway might involve in the pathogenesis of SLE^44^. This highly connected PPI cluster could be conceived as the core of the formula.

Additionally, this core PPI cluster was connected to PPI branches that included different disease protein clusters. For example, one PPI branch includes two clusters (Fig. 3b): cluster1: collagen, type II, alpha 1 (STL1), complement component 1, r subcomponent (C1R), complement component 1, s subcomponent (C1S), complement component 1, q subcomponent, B chain (C1QB), complement component 1, q subcomponent, C chain (C1QG) and complement component 1, q subcomponent, A chain (C1QA); and cluster2: transforming growth factor, beta 1 (TGF-beta), beta-glycan, transforming growth factor, beta receptor II (TGFbeta-RII), transforming growth factor, beta receptor 1 (TGFR-1), transforming growth factor, beta 3 (TGFbeta3) and suppressor of cytokine signaling 6 (STATI4). The proteins in cluster1 were involved in the immune response, innate immunity and the complement pathway, which were closely correlated with the immune system. In details, reduced central tolerance to STL1 might induce and aggravate arthritis caused by cross-reactive autoantibody production^45^, and C1Q deficiency was prevalence in SLE patients while Anti-C1q antibodies was identified as a marker in SLE patients^46^,^47^. The proteins in cluster2 stimulated invasion and metastasis during carcinogenesis and promoted many pathological fibrotic diseases when overstimulated^48^. To be specific, STATI4 was associated with the development of SLE by inhibiting growth of NK cells^49^,^50^. One target (TGFbeta) linking the two disease protein clusters together could be the key protein connecting the two functional modules. Indeed, autoimmunity was triggered as a result of the decreased immunosuppressive effect induced by depressed TGFbeta levels in patients with SLE^51^.

### Interactions between common targets and SLE disease proteins

For common targets, we discovered that they closely interacted with essential targets and SLE disease proteins (Supplementary Fig. S2), however, their network properties were different. The average degree of common targets (2.7) was smaller than that of essential targets (4.9) and SLE disease proteins (3.3); the average degree of essential targets was the highest of the three. A total of 45% of common targets with low degrees (<3) interacted with specific essential targets or SLE disease proteins. For instance, the essential target chemokine receptor 2 (IL8RB) interacted with 10 common targets (guanine nucleotide binding protein [G protein], alpha inhibiting activity polypeptide 2 [H_LUCA16.1], chemokine ligand 3 [SCYB3], chemokine ligand 1 [SCYB1], guanine nucleotide binding protein alpha 15 [GNA16], chemokine ligand 2 [SCYB2], chemokine ligand 5 [SCYB5], chemokine receptor 1 [IL8RBA], guanine nucleotide binding protein alpha 14 [GNA14], pro-platelet basic protein [THBGB1] and chemokine ligand 5 [SCYB6]) (Supplementary Figure S3a). IL-8RBA was involved in the augmentation of tissue injury in SLE during inflammatory responses by cooperating with FcgammaRIIa and enhancing polymorphonuclear leukocyte (PMN) recruitment in the presence of anti-endothelial cell antibodies (AECAs), which were commonly detected in diseases associated with vascular injury, including SLE^52^. Seven of the 10 common targets (guanine nucleotide binding protein [G protein], alpha inhibiting activity polypeptide 2 [H_LUCA16.1], chemokine ligand 3 [SCYB3], chemokine ligand 1 [SCYB1], guanine nucleotide binding protein alpha 15 [GNA16], chemokine ligand 2 [SCYB2], chemokine ligand 5 [SCYB5], chemokine receptor 1 [IL8RBA], guanine nucleotide binding protein alpha 14 [GNA14], pro-platelet basic protein [THBGB1] and chemokine ligand 5 [SCYB6]) participated in the chemokine signaling pathway that was involved in SLE pathogenesis^53^. The function of most the proteins in this module was about chemokine, indicating this module could be considered as chemokine module. Another essential target (tyrosine 3-monooxygenase [YWHAA]) interacted with 15 common targets (centromere protein C [MIF2], cyclin-dependent kinase inhibitor 1B [P27KIP1], uncoupling protein 2 [UCPH], microtubule-associated protein tau [TAU], uncoupling protein 3 [SLC25A9], tyrosine hydroxylase [TYH], protein tyrosine phosphatase, non-receptor type 3 [PTPH1], insulin receptor substrate 2 [IRS-2], insulin receptor [HHF5], insulin receptor substrate 1 [HIRS-1], Raf-1 proto-oncogene, serine/threonine kinase [C-Raf], protein kinase C, delta [nPKC-delta], ABL proto-oncogene 1, non-receptor tyrosine kinase [v-abl], cell division cycle 25C [PPP1R60] and p60-Src) (Supplementary Figure S3b). Four of the 15 common targets were members of the PI3K-AKT signaling pathway. The PI3K/Akt/mTOR signaling pathway was essential to cellular proliferation and growth signaling and was correlated with autoimmune diseases due to its activation in lymphocytes that developed features of systemic autoimmunity^54^. These two examples demonstrate that the low-degree, common targets mostly interact with disease or essential targets, suggesting they mainly play supporting roles in the formula.

In addition to the low-degree common targets, common targets with higher degrees (≥3) could also play essential roles in connecting SLE-related proteins. Common target epidermal growth factor receptor (mENA) interacted with 16 essential targets (estrogen receptor 1 [NR3A1], signal transducer and activator of transcription 3 [HIES], signal transducer and activator of transcription 1, 91kDa [STAT91], Fas cell surface death receptor [TNFRSF6], intercellular adhesion molecule 1 [P3.58], cyclin-dependent kinase inhibitor 2A [P14], small nuclear ribonucleoprotein D2 polypeptide 16.5kDa [SM-D2], erb-b2 receptor tyrosine kinase 3 [p85-sErbB3], protein tyrosine phosphatase, non-receptor type 2 [TCPTP], signal transducer and activator of transcription 5 [STAT5], protein tyrosine phosphatase, non-receptor type 6 [SHP1], Janus kinase 2 [THCYT3], protein kinase, cAMP-dependent, regulatory, type I, alpha [TSE1], S-phase kinase-associated protein 2, E3 ubiquitin protein ligase [P45], angiotensin II receptor, type 1 [HAT1R] and estrogen receptor 1 [NR3A1]) (Fig. 3c). These essential targets were involved in SLE pathogenesis; moreover, genetic variants of NR3A1 (estrogen receptor, ranked as the highest degree of the 16 essential targets) might influence its susceptibility for involvement in pathogenesis^55^. THCYT3, SHP1 and STAT91 influenced the pathogenesis of SLE through the JAK-STAT1 signaling pathway^56^, while mENA (usually known as a direct regulator of microfilament polymerization and bundling) promoted metastasis in various cancers^57^. Another common target, MYD88B, interacted with 9 essential targets (interferon regulatory factor 5 [SLEB10], toll-like receptor 7 [TLR7-like], interleukin 1 receptor, type I [P80], toll-like receptor 5 [TIL3], toll-like receptor 4 [TOLL], toll-like receptor 4 [TIL4], Fas (TNFRSF6)-associated via death domain [MORT1], interleukin-1 receptor-associated kinase 1 [pelle] and interferon regulatory factor 7 [IRF7H]) (Fig. 3d); MYD88B participated in the TLR signaling pathway with 8 of these targets (excluding P80). SLEB10 deficiency inhibited autoantibody production and ameliorates SLE disease likely due to its effects on TLR7 and TLR9^58^. This example presents a potential key role for MYD88B in the TLR signaling pathway during SLE treatment. One final complicated example was about the interactions between 84 common targets and 24 essential targets (Supplementary Figure S4). Common target p60-src interacted with all 24 essential targets, demonstrating its essential role in this cluster. P60-src (Csk) was critical to multiple maturation and activation steps in B cells and physically interacted with the intracellular phosphatase encoded by protein tyrosine phosphatase, non-receptor type 22 (PTPN22) to form the Lyp-Csk complex that was capable of modifying the activation state of downstream SRC proto-oncogene, non-receptor tyrosine kinase (Src kinases) (e.g. Lyn in lymphocytes) and increasing susceptibility to SLE^58^. Overall, these examples demonstrate that the non-key high-degree targets connect different functional modules related to SLE biological processes, suggesting their important functions in SLE treatment.

### Evaluation of targets with SLE functional modules

Analysis of patient transcriptional profiles offers a means to investigate mechanisms relevant to human diseases on a genome-wide scale. Using genome-wide expression profiling, Damien Chaussabel and his colleagues identified transcriptional modules based on genes co-expressed in multiple disease data sets. They constructed peripheral blood mononuclear cell (PBMC) transcriptional modules focusing on small sets of coordinately expressed transcripts. Using the “data-driven” and 3-round module-selection process, they finally obtained 28 functional modules, including 3,485 proteins, which were then used to map transcriptional changes between patients and healthy subjects. A total of 235 SMBJT targets were included in these 28 functional modules (Supplementary Table S5). Among the 28 modules, 11 modules were related to SLE; a total of 9 out of the 11 modules were direct correlated to immune system. For example, M1.1 included genes encoding for immunoglobulin (Ig) chains and the plasma cell marker. Other than healthy individuals and patients suffering from other systemic autoimmune disease, light chain Igk and Igλ were expressed simultaneously in B cells from SLE patients, and the allelic inclusion exhibited high frequencies of having lupus nephritis^59^. Nevertheless, inhibition of long-lived plasma cells was involved in several therapeutic strategies of SLE^60^.

Furthermore, we identified targets of SMBJT that were significantly enriched in three modules (M1.5, M2.6 and M3.3). The functions of the proteins in these three modules were related to the myeloid lineage (M1.5 and M 2.6) and Inflammation II (M3.3), suggesting the involvement of SMBJT targets in these major biological processes. Genes in M1.5 encode proteins expressed in myeloid-lineage cells (CD86 molecule, CD163 molecule and Fc fragment of IgG, low affinity IIa, receptor [FCGR2A]), proteins involved in pathogen recognition (CD14 molecule, toll-like receptor 2 [TLR2] and MYD88) and TNF-family members (tumor necrosis factor receptor superfamily, member 1B [TNFR2] and tumor necrosis factor superfamily, member 13b [BAFF]). Genes in M2.6 (IGTB2/CD18, Lymphotoxin beta receptor, Myeloid related proteins 8/14, and Formyl peptide receptor 1) were expressed in myeloid-lineage cells such as monocytes and neutrophils. Genes in M3.3 encode proteins (interleukin 18 [IL-18], arachidonate 5-lipoxygenase [ALOX5], alanyl aminopeptidase [ANPEP], acyloxyacyl hydrolase [AOAH], heme oxygenase 1 [HMOX1] and serpin peptidase inhibitor, clade B, member 1 [SERPINB1]) that were induced by inflammation or lysosomal enzymes (palmitoyl-protein thioesterase 1 [PPT1], cathepsin B/S [CTSB/S], sialidase 1 [NEU1], N-acylsphingosine amidohydrolase 1 [ASAH1], lysosomal-associated membrane protein 2 [LAMP2] and calpastatin [CAST]).

## Discussion

SMBJT is a TCM formula that is widely used for SLE treatment. In this study, we evaluated the potential molecular mechanism of SMBJT. Functional analysis of the 1,267 targets of SMBJT highlighted the importance of these targets in regulation, signaling and metabolism. 96 essential and 1,171 common targets of this formula were identified, and their relationships with SLE disease genes in a PPI network were investigated. Hub proteins were identified in network clusters to play important roles in this formula. The small number of targets and SLE disease proteins that do not interact with one another could contribute to SMBJT’s side effects and treatment failure in a small number of clinical cases. The biological function of the SMBJT targets identified in this work will help us to understand how this TCM formula treats the complex disease SLE.

**Figure 4:**
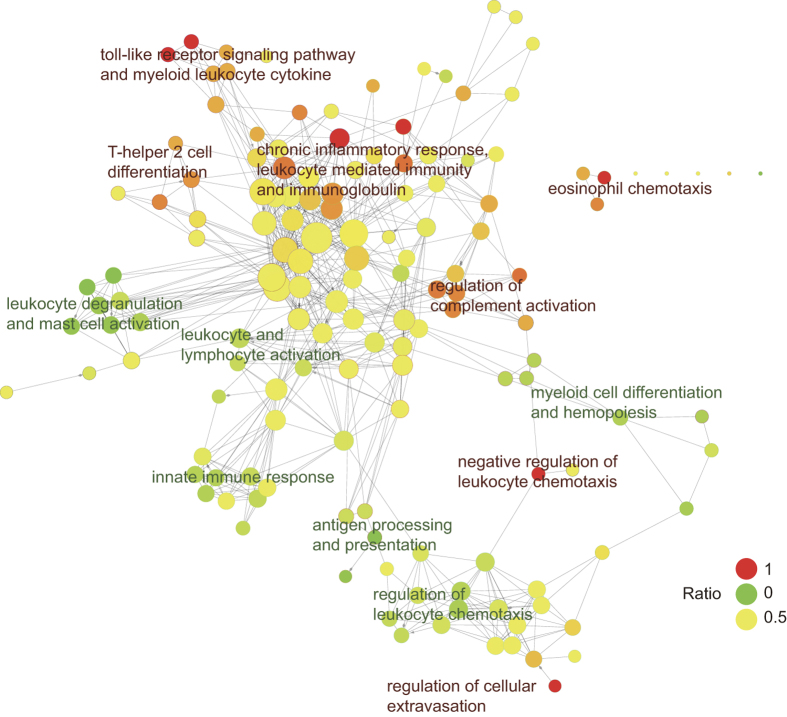
The ratio of essential targets and common targets in immune system processes. The network was generated with ClueGO in Cytoscape.

Considering the significance of immune system processes in the formula targets, we compared the ratio of essential targets and common targets in the whole immune system to discover the functional relationships between two types of targets (Fig. 4). We found that key targets were involved in the core component of the immune upstream regulation processes significantly correlated with SLE, e.g. toll-like receptor signaling pathway, myeloid leukocyte cytokine, chronic inflammatory response, leukocyte mediated immunity, immunoglobulin, etc. The chronic inflammatory response was involved in the development and prognosis of SLE^61^. Fragments of immunoglobulins were significantly corrected with SLE disease activity^62^, and complement receptor of the immunoglobulin superfamily could diminish inflammation and reversed established bone destruction^63^. On the other hand, common targets normally participated in the peripheral immune processes that were more specific to leukocyte, e.g. leukocyte degranulation, leukocyte chemotaxis, etc. We also discover that several immune processes dominated by essential targets served as the important regulators in the peripheral immune processes, e.g. negative regulation of leukocyte chemotaxis and regulation of cellular extravasation. The overview of targets in immune system suggests essential and common targets cover the most important processes with distinct immune function, and indicating the SMBJT might provide more systematically therapy for SLE patients in TCM.

In the further investigation of the relationships between essential and common targets in toll-like receptor signaling pathway (Supplementary Figure S5) and chemokine signaling pathway (Supplementary Figure S6), we discovered that the common targets largely filled the empty between essential targets and composed a more complete target path. In toll-like receptor signaling pathway (Supplementary Figure S5), the common target, MYD88 filled the blank step between two essential targets (TLR2 and Fas-associated via death domain [FADD]) that formed a complete signaling path lead to apoptosis. Thus, we believe that in the treatment of complex disease, SMBJT has more drug targets (especially common targets that were not defined as SLE disease gene before) and provides more systematical therapy for patients. This more genome-wide TCM drug mechanism could also avoid the palliatives in single target medical service for the complex autoimmune disease.

Traditional research into the mechanism behind TCM formulas was based on single-ingredient and single-target models. However, the mechanism should be investigated using complex multi-target interactions, which has proven challenging for researchers. The rapid development of bioinformatics offers an alternative approach for the study of the mechanisms of TCM formulas using multi-level data. The method we proposed here provides a new way to comprehensively understand the therapeutic effects of TCM formulas in SLE treatment. This work could serve as a good example for the studies of the mechanisms of TCM formulas on other complex diseases.

## Materials and Methods

### Data collection

The TCMID database (TCMID, http://www.megabionet.org/tcmid/) contains a large amount of information concerning formulas and their herbal ingredients^12^. We retrieved the ingredients of six herbs and their protein targets from TCMID for further analysis. The STITCH defined targets of ingredients with confidence ranges for data scores (low confidence: scores <0.4; medium: 0.4 to 0.7; high: >0.7)^17^. Based on these scores, we chose 1,267 high confident targets with comprehensive scores >0.7. Genes associated with SLE were collected from three databases: OMIM (http://www.omim.org)^18^, GAD (http://geneticassociationdb.nih.gov/)^64^ and KEGG (http://www.kegg.jp)^65^.

### Gene ontology and pathway enrichment analysis

DAVID Bioinformatics Resources 6.7 (http://david.abcc.ncifcrf.gov/)^66^, which represents a comprehensive set of functional annotation tools for understanding the biological meanings behind large gene datasets, were used to perform Gene Ontology (GO) enrichment analysis for the 1,267 genes targeted by SMBJT. DAVID was also applied to conduct pathway enrichment analysis for the same 1,267 gene products to identify associations between SLE and SMBJT. Enriched GO terms and pathways were defined as those with adjusted p-values <0.05.

### Network construction

Based on the protein-protein interaction data in HPRD (http://www.hprd.org/)^67^ and STRING (http://string-db.org/)^68^, we constructed a protein-protein interaction (PPI) network for the 1,267 target proteins of SMBJT and the SLE susceptibility genes to obtain the hub effective proteins. The networks were visualized with Cytoscape^69^.

### Module enrichment analysis

We adopted the 28 functional modules identified by Damien Chaussabel and his colleagues^22^. A total of 11 modules contained differentially expressed genes related to SLE. Among the 4,742 transcripts contained in the 28 functional modules, 3,485 have been annotated. We compared the 3,485 proteins with the target proteins of SMBJT and identified 235 common genes (Supplementary Table S5). We selected modules containing at least one SMBJT target gene. Next, we performed module enrichment analysis for the 28 functional modules.

The enrichment analysis was designed to filter out differences in expression levels of gene sets between two or more groups (especially enriched gene sets). Common gene enrichment analysis methods can be summarized into two categories: the bottom-up method and the top-down method. The bottom-up method is the most widely used; this method starts with single-gene analysis and then continues with further analyses based on biological information, such as GO annotation gene sets. Common bottom-up analysis methods include Fisher’s exact test, gene enrichment analysis, gene enrichment parameter analysis and gene set analysis^70^. Here, we chose Fisher’s exact test. Let Gc denote the set of extracted proteins in component c and G denote the set of proteins in a functional unit (functional module). Let r = |Gc|, k = |G|, z = |Gc 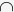 G| and l be the total number of proteins in the whole dataset. We assumed that z followed a hypergeometric distribution. The probability of observing an intersection of size z between G and Gc was computed as equation (1). The enrichment score of component c was defined in equation (2). Enrichment of the target proteins of TCM in the identified functional modules (adjusted p-values < 0.05) indicated that these proteins contributed to the therapeutic mechanism of TCM.


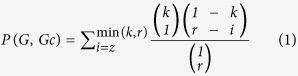



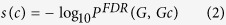


## Additional Information

**How to cite this article**: Huang, L. *et al*. A Systems Biology-Based Investigation into the Pharmacological Mechanisms of Sheng-ma-bie-jia-tang Acting on Systemic Lupus Erythematosus by Multi-Level Data Integration. *Sci. Rep*. **5**, 16401; doi: 10.1038/srep16401 (2015).

## Supplementary Material

Supplementary Information

## Figures and Tables

**Table 1:** The top 20 KEGG pathways with p-values < 0.05 generated by DAVID.

KEGG pathway	Counts ofgenes	p-value	Class
Chemokine signaling pathway	55	5.80E-06	Organismal Systems; Immune system
Toll-like receptor signaling pathway	35	9.70E-06	Organismal Systems; Immune system
Adipocytokine signaling pathway	34	2.30E-10	Organismal Systems; Endocrine system
PPAR signaling pathway	33	3.00E-09	Organismal Systems; Endocrine system
Drug metabolism	23	1.00E-07	Metabolism; Xenobiotics biodegradation and metabolism
Metabolism of xenobiotics by cytochrome P450	28	1.10E-07	Metabolism; Xenobiotics biodegradation and metabolism
Drug metabolism	27	1.10E-06	Metabolism; Xenobiotics biodegradation and metabolism
Fatty acid metabolism	23	1.80E-08	Metabolism; Overview
Retinol metabolism	38	6.60E-18	Metabolism; Metabolism of cofactors and vitamins
Pyruvate metabolism	24	2.60E-09	Metabolism; Carbohydrate metabolism
Citrate cycle (TCA cycle)	20	1.50E-08	Metabolism; Carbohydrate metabolism
Propanoate metabolism	19	2.30E-07	Metabolism; Carbohydrate metabolism
Glycolysis / Gluconeogenesis	26	0.000002	Metabolism; Carbohydrate metabolism
Tryptophan metabolism	19	0.000015	Metabolism; Amino acid metabolism
Tyrosine metabolism	21	4.20E-06	Metabolism; Amino acid metabolism
Arginine and proline metabolism	22	0.000033	Metabolism; Amino acid metabolism
Cytokine-cytokine receptor interaction	69	0.000021	Environmental Information Processing; Signaling molecules and interaction
Neuroactive ligand-receptor interaction	133	7.90E-42	Environmental Information Processing; Signaling molecules and interaction
