# Supplementary material for: A Systems Biology-Based Investigation into the Pharmacological Mechanisms of Sheng-ma-bie-jia-tang Acting on Systemic Lupus Erythematosus by Multi-Level Data Integration
Source: Sci Rep. 2015 Nov 12;5:16401. doi: 10.1038/srep16401 (PMC4642335; doi:10.1038/srep16401)
Supplement: Supplementary Information [file srep16401-s1.pdf]

A Systems Biology-Based Investigation into the Pharmacological Mechanisms of Sheng-ma-bie-jia-tang Acting on Systemic Lupus Erythematosus by Multi-Level Data Integration

Lin Huang[1, #], Qi Lv[2, 3, #], Fenfen Liu[1, #], Tieliu Shi[2, 4, *], Chengping Wen[1, *]

[1]TCM Clinical Basis Institute, Zhejiang University of Chinese Medicine, 548 Binwen Road, Hangzhou, Zhejiang, 310000, China. [2]Center for Bioinformatics and Computational Biology, and the Institute of Biomedical Sciences, School of Life Science, East China Normal University, 500 Dongchuan Road, Shanghai, 200241, China. [3]School of Finance and Statistics, East China Normal University, 500 Dongchuan Road, Shanghai, 200241, China. [4]Biological Targeting Diagnosis and Therapy Research Center, Guangxi Medical University, Nanning, Guangxi, China. [#]These authors contributed equally to this work. Correspondence and requests for materials should be addressed to T.S. (email: tieliushi01@gmail.com) and C.W. (email: wengcp@163.com).

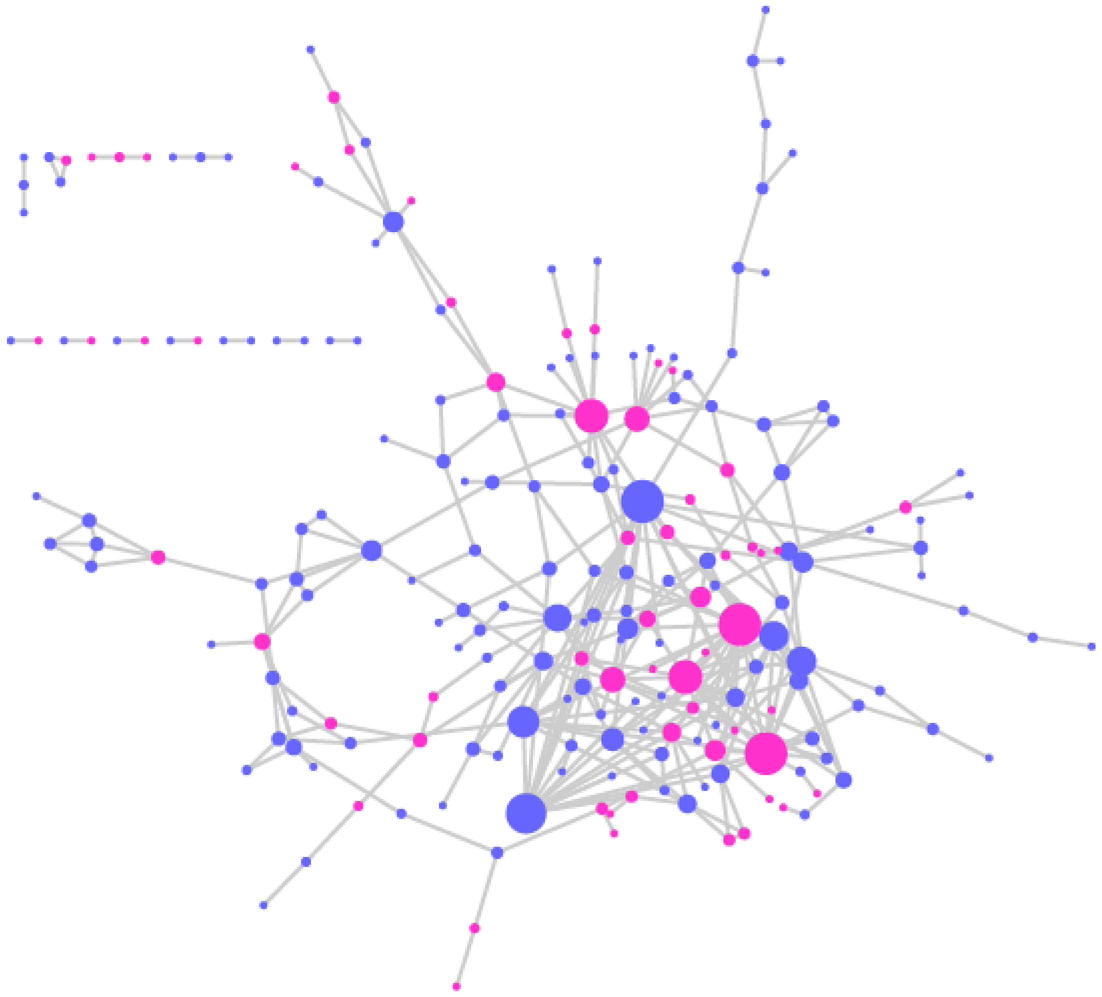

Supplementary Figure S1 PPI network of SLE disease genes and essential targets. Blue nodes, the products of SLE disease genes; red nodes, the products of essential targets.

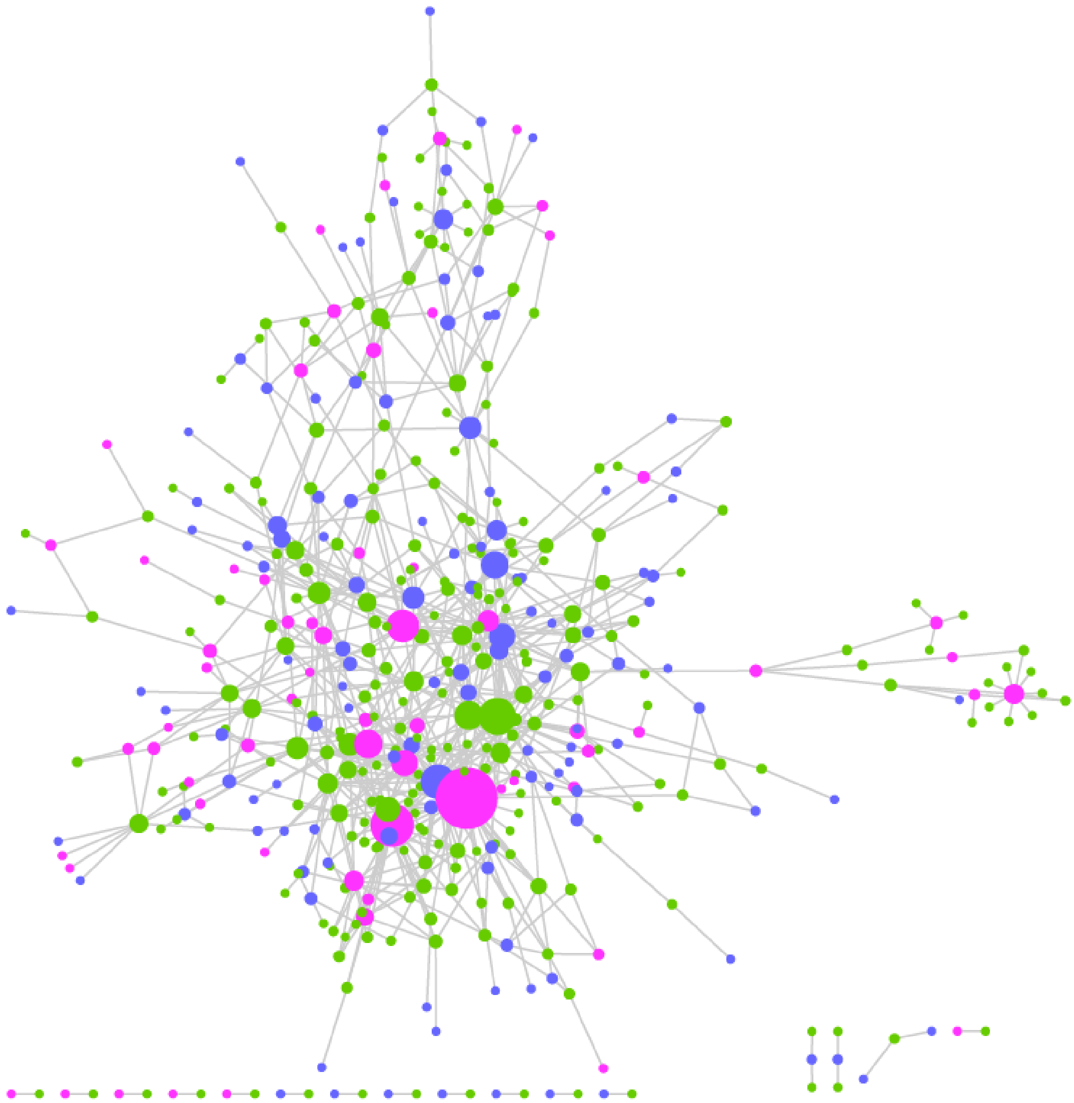

Supplementary Figure S2 PPI network of SLE disease genes, essential targets and common targets. Blue nodes, the products of SLE disease genes; red nodes, the products of essential targets; green nodes, the products of common targets.

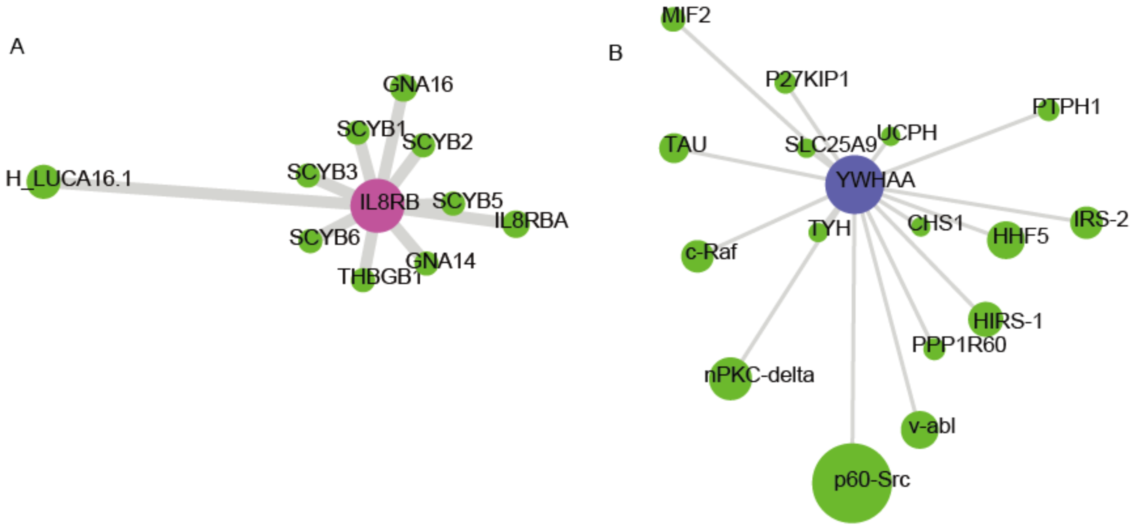

Supplementary Figure S3 Common target network of IL-8RB and YWHAA. (a) Sub-network of IL-8RB and low-degree common targets. (b) Sub-network of YWHAA and low-degree common targets. Blue nodes, the products of SLE disease genes; red nodes, the products of essential targets; green nodes, the products of common targets.

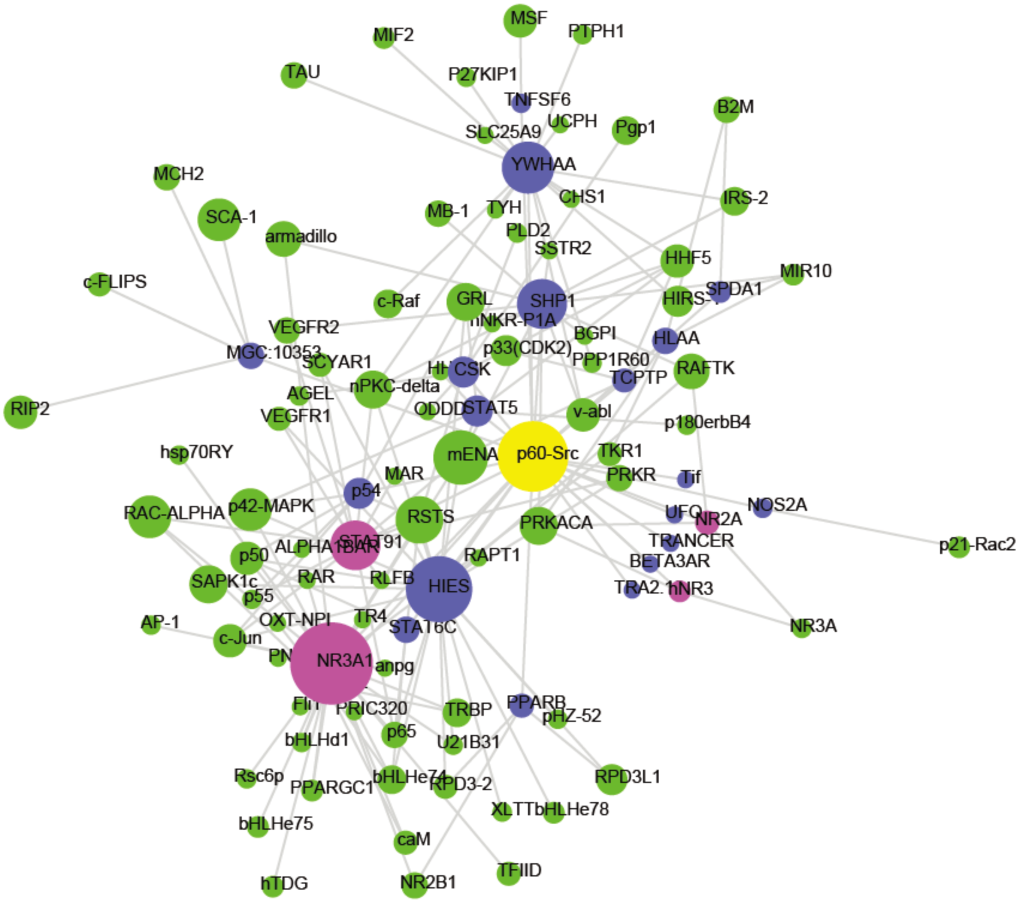

Supplementary Figure S4 PPI sub-network of the high-degree, common target p60-Src. Blue nodes, the products of SLE disease genes; red nodes, the products of essential targets; green nodes, the products of common targets.

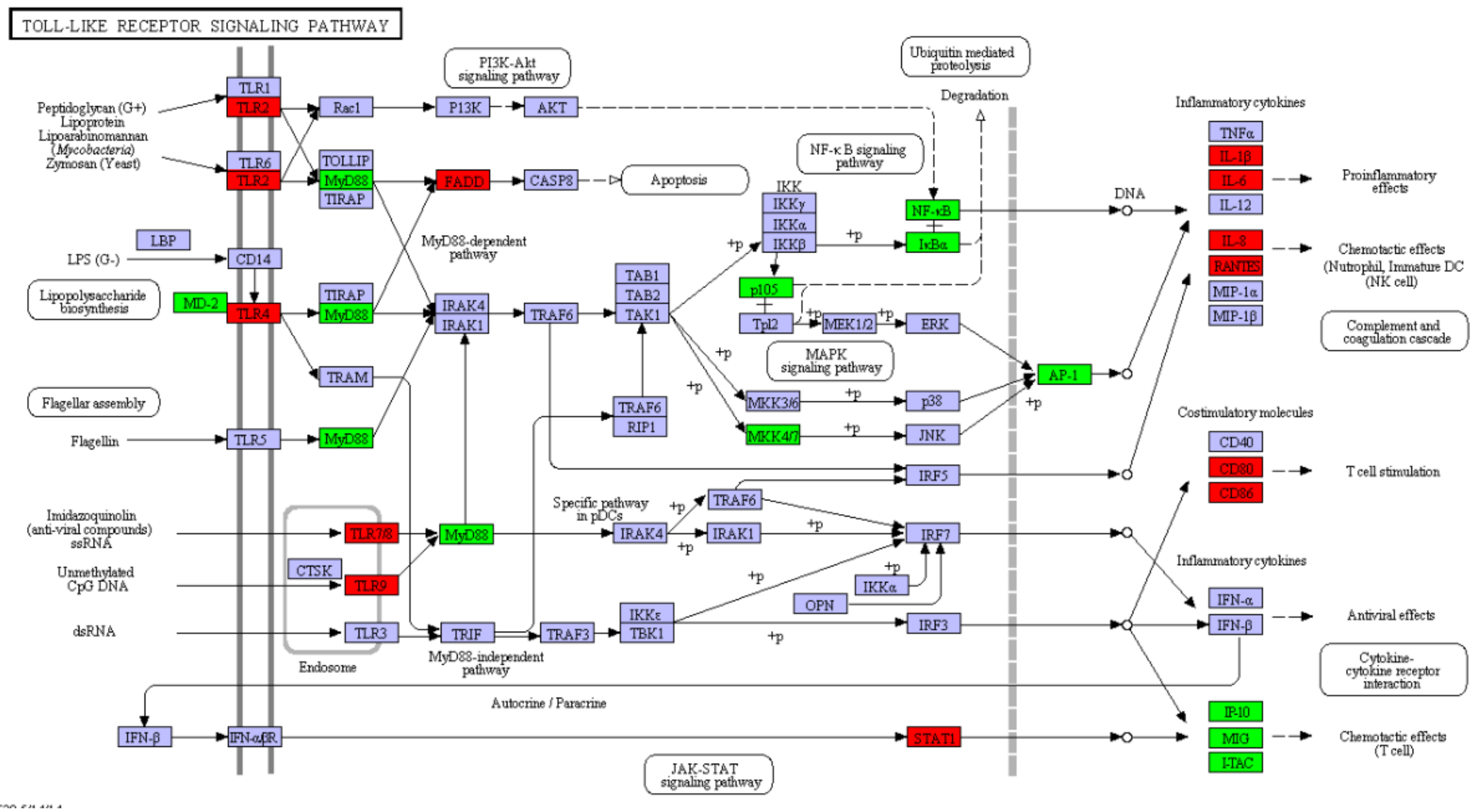

Supplementary Figure S5 The participation of essential and common targets in toll-like receptor signaling pathway. The graph was generated with KEGG online tool. Red: essential targets; green: common targets; purple: other protein in the pathway.

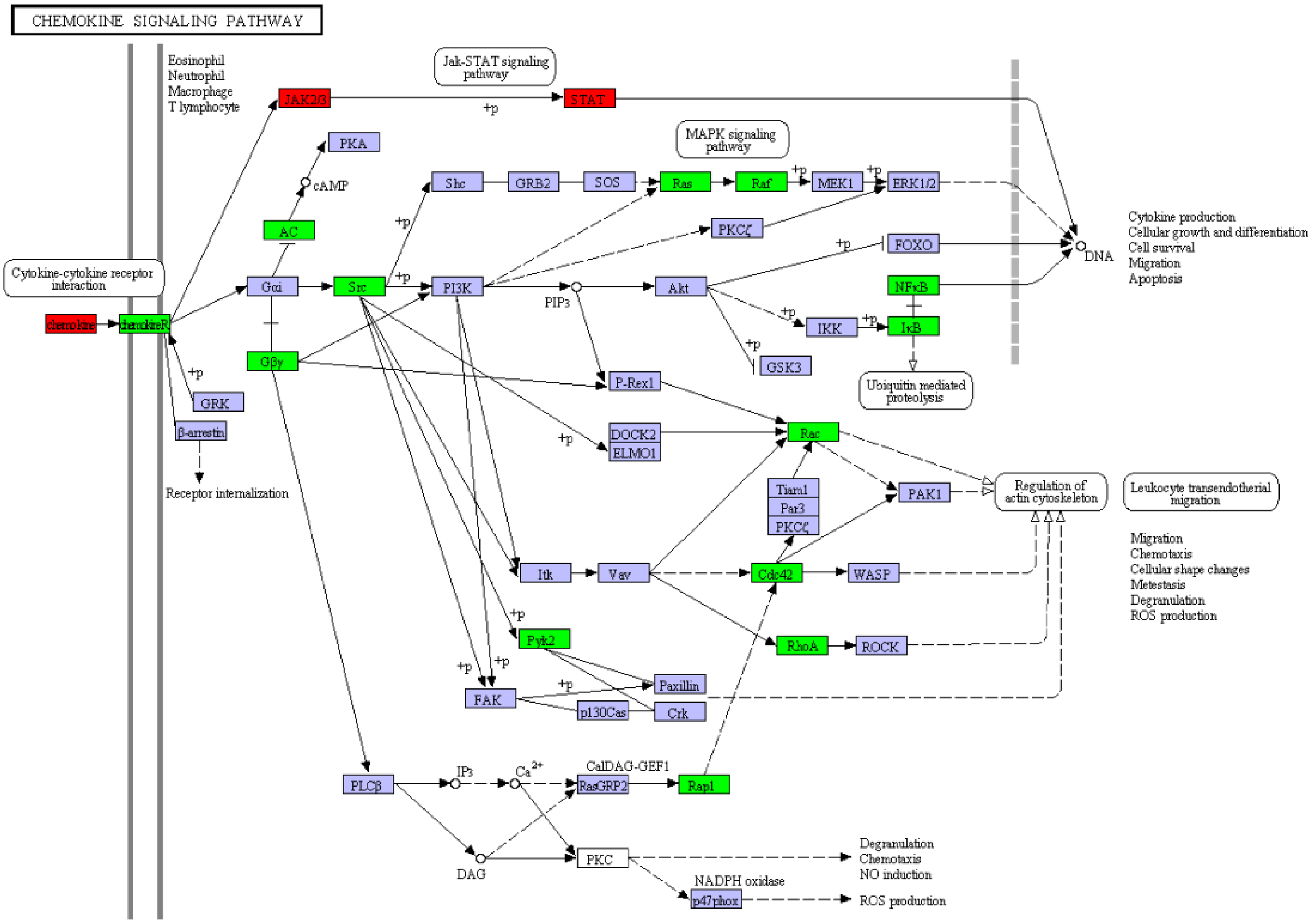

Supplementary Figure S6 The participation of essential and common targets in chemokine signaling pathway. The graph was generated with KEGG online tool. Red: essential targets; green: common targets; purple: other protein in the pathway.

Supplementary Table S1 Detailed description of the herbs and ingredients in SMBJT.

**Herb**	**ingredient**
Cimicifuga foetida	cimicilen
Cimicifuga foetida	3-ket-o-24-epi-7, 8-didehydrocimigenol
Cimicifuga foetida	cimigenol-3-o-beta-d-xylopyranoside
Cimicifuga foetida	hespereticacid
Cimicifuga foetida	cimifoetiside iii
Cimicifuga foetida	carlumine
Cimicifuga foetida	racemoside
Cimicifuga foetida	dahurinol
Cimicifuga foetida	cimiside e
Cimicifuga foetida	cimiside a
Cimicifuga foetida	cycloartenol
Cimicifuga foetida	cimicifugcacid
Cimicifuga foetida	cimigenol xyloside
Cimicifuga foetida	caffeicacid
Cimicifuga foetida	beta-sitiosterol
Cimicifuga foetida	adlumidine
Cimicifuga foetida	cimiside b
Cimicifuga foetida	isoferulic acid
Cimicifuga foetida	n-salicylidene-salicylamine
Cimicifuga foetida	salicylic acid
Cimicifuga foetida	visamminol
Cimicifuga foetida	6-o-e-feruloylajugol
Cimicifuga foetida	24-epi-7, 8-didehydrocimigenol
Cimicifuga foetida	12β-acetoxycimigenol-3-o-β-d-xylopyranoside
Cimicifuga foetida	norcimifugin
Cimicifuga foetida	cimioifugamid i
Cimicifuga foetida	7,8-didehydro-27-deoxyactein
Cimicifuga foetida	caffeic acid
Cimicifuga foetida	shengmanol xyloside
Cimicifuga foetida	visnagin
Cimicifuga foetida	25-o-acetyl-7, 8-didehydrocimigenol
Cimicifuga foetida	ferulic acid
Cimicifuga foetida	25-o-acetylcimigenol-3-o-beta-d-xylopyranoside
Cimicifuga foetida	macrotin

Cimicifuga foetida	isoflavone formononeline
Cimicifuga foetida	caffeic acid dimethyl ether
Cimicifuga foetida	7, 8-didehydrocimigenol
Cimicifuga foetida	ferulicacid
Cimicifuga foetida	cimigenol
Cimicifuga foetida	bicuculline
Cimicifuga foetida	23-o-acetylshengmanol 3-o-α-l-arabinopyrano-side
Cimicifuga foetida	2', 4'-o-diacetyl-24-epi-7, 8didehydrocimigenol-3-xyloside
Carapax Trionycis	coloagen
Carapax Trionycis	keratin
Carapax Trionycis	vitamin d
Glycyrrhiza uralensis	methyl-n-butyl-uralsaponin a esters
Glycyrrhiza uralensis	licoleafol
Glycyrrhiza uralensis	licopyranocoumarin
Glycyrrhiza uralensis	licoricesaponin a3
Glycyrrhiza uralensis	licofuranocoumarin
Glycyrrhiza uralensis	licoisoflavone
Glycyrrhiza uralensis	ononin
Glycyrrhiza uralensis	8-methoxy-5-o-glucoside flavone
Glycyrrhiza uralensis	3'-methoxyglabridin
Glycyrrhiza uralensis	licoricesaponin b2
Glycyrrhiza uralensis	licoricesaponin c2
Glycyrrhiza uralensis	nicotiflorin
Glycyrrhiza uralensis	gmelofuran
Glycyrrhiza uralensis	ferulic acid
Glycyrrhiza uralensis	glyzaglabrin
Glycyrrhiza uralensis	glycyrrhiza-flavonol a
Glycyrrhiza uralensis	tetrahydropalmatine
Glycyrrhiza uralensis	hispidulin
Glycyrrhiza uralensis	3-o-acetyl-glycyrrhetinicacid
Glycyrrhiza uralensis	glabrolide
Glycyrrhiza uralensis	glycyrrhetinic acid
Glycyrrhiza uralensis	2,4,4'-trihydroxychalcone
Glycyrrhiza uralensis	gancaonin p-3'-methylether
Glycyrrhiza uralensis	gancaonin x
Glycyrrhiza uralensis	sigmoidin b
Glycyrrhiza uralensis	licoricesaponine j2
Glycyrrhiza uralensis	neoliquiritin

Glycyrrhiza uralensis	3,4-dicaffeoyl-5-(3-hydroxy-3-methyl) glutaroyl quinic acid
Glycyrrhiza uralensis	licoricesaponine k2
Glycyrrhiza uralensis	n-tricosane
Glycyrrhiza uralensis	isoononin
Glycyrrhiza uralensis	8-methyl-10-hydroxylycoctonine
Glycyrrhiza uralensis	(e)-1-[2,4-dihydroxy-3-(3-methyl-2-butenyl)phenyl]-3-(2,2-dimethyl-8-hydroxy-2h-benzo-pyran-6-yl)-2-propen-1-one
Glycyrrhiza uralensis	methyl-24-hydroxyglycyrrhetate
Glycyrrhiza uralensis	(e)-1-[2,4-dihydroxy-3-(3-methyl-2-butenyl)phenyl]-3-(4-hydroxy-3-[3-methyl-2-butenyl)phenyl]-2-propen-1-one
Glycyrrhiza uralensis	methylglycyrrhetate
Glycyrrhiza uralensis	methylglyoxal
Glycyrrhiza uralensis	licorisoflavan a
Glycyrrhiza uralensis	licoricone
Glycyrrhiza uralensis	isoliquiriligenin
Glycyrrhiza uralensis	kanzonol l
Glycyrrhiza uralensis	kanzonol k
Glycyrrhiza uralensis	18alpha-glycyrrhetinic acid
Glycyrrhiza uralensis	licoricesaponin e2
Glycyrrhiza uralensis	licoricesaponin d3
Glycyrrhiza uralensis	licoricesaponin g2
Glycyrrhiza uralensis	licoricesaponin f3
Glycyrrhiza uralensis	licoricesaponin j2
Glycyrrhiza uralensis	licoricesaponinh2
Glycyrrhiza uralensis	licoricidin
Glycyrrhiza uralensis	licoricesaponin k2
Glycyrrhiza uralensis	3-hydroxyglabrol (ii)
Glycyrrhiza uralensis	liquiritin
Glycyrrhiza uralensis	liquiritigenin-7,4'-diglucoside
Glycyrrhiza uralensis	liquiritigenin4'-o-β-d-apio-d-furanosyl(1→2)-β-d-glucopyranoside
Glycyrrhiza uralensis	liquiritigenin
Glycyrrhiza uralensis	gancaonin f
Glycyrrhiza uralensis	gancaonin i
Glycyrrhiza uralensis	gancaonin b
Glycyrrhiza uralensis	gancaonin c
Glycyrrhiza uralensis	gancaonin d
Glycyrrhiza uralensis	gancaonin e

Glycyrrhiza uralensis	(e)-1-[2,4-dihydroxy-3-(3-methyl-2-butenyl)phenyl]-3-(4-hydroxy-3-[3-methyl-2-butenyl) phenyl]-2-propen-1-one
Glycyrrhiza uralensis	gancaonin a
Glycyrrhiza uralensis	rutin
Glycyrrhiza uralensis	ruvoside
Glycyrrhiza uralensis	umbelliferone
Glycyrrhiza uralensis	narwedine
Glycyrrhiza uralensis	glycyrrhizin
Glycyrrhiza uralensis	isogosferol
Glycyrrhiza uralensis	isoglycyrol
Glycyrrhiza uralensis	liquiriligenin
Glycyrrhiza uralensis	3'-(γ,γ-dimethylallyl)-kievitone
Glycyrrhiza uralensis	formononetin-7-glucoside
Glycyrrhiza uralensis	isoquercitrin
Glycyrrhiza uralensis	schaftoside
Glycyrrhiza uralensis	uralstilbene
Glycyrrhiza uralensis	phaseollinisoflavan
Glycyrrhiza uralensis	phebalosin
Glycyrrhiza uralensis	uralenol
Glycyrrhiza uralensis	uralenneoside
Glycyrrhiza uralensis	uralsaponin a
Glycyrrhiza uralensis	uralenol-3-methylether
Glycyrrhiza uralensis	uralenin
Glycyrrhiza uralensis	uralene
Glycyrrhiza uralensis	5,6,7,8-tetrahydro-4-methylquinoline
Glycyrrhiza uralensis	neoisopulegol
Glycyrrhiza uralensis	neoisoliquiritin
Glycyrrhiza uralensis	astragalin
Glycyrrhiza uralensis	5,6,7,8-tetrahydro-2, 4-dimethylquinoline
Glycyrrhiza uralensis	monoammonium glycyrrhizinate
Glycyrrhiza uralensis	β-sitosterol
Glycyrrhiza uralensis	tetrahydroharmine
Glycyrrhiza uralensis	dibutyl uralsaponin a ester
Glycyrrhiza uralensis	isoschaftoside
Glycyrrhiza uralensis	4'-o-methylglabridin
Glycyrrhiza uralensis	glycyrrhizic acid
Glycyrrhiza uralensis	isoramanone
Glycyrrhiza uralensis	glycyphyllin
Glycyrrhiza uralensis	ethyl-n-buthy-uralsaponin a esters

Glycyrrhiza uralensis	hispaglabridin b
Glycyrrhiza uralensis	hispaglabridin a
Glycyrrhiza uralensis	6,8-bis(c-β-glucosyl)-apigenin
Glycyrrhiza uralensis	3-hydroxyglabrol
Glycyrrhiza uralensis	urea
Glycyrrhiza uralensis	corylifolinin
Glycyrrhiza uralensis	ganoderic acid a
Glycyrrhiza uralensis	isotrifoliol
Glycyrrhiza uralensis	isotrilobine
Glycyrrhiza uralensis	berniarin
Glycyrrhiza uralensis	isoliquiritin
Glycyrrhiza uralensis	isolobelanine
Glycyrrhiza uralensis	isoliquiritigenin
Glycyrrhiza uralensis	uralsaponin b
Glycyrrhiza uralensis	gamma-sitosterol
Glycyrrhiza uralensis	ononitol
Glycyrrhiza uralensis	sinapic acid
Glycyrrhiza uralensis	isoorientin
Glycyrrhiza uralensis	neohancoside a
Glycyrrhiza uralensis	neouralenol
Glycyrrhiza uralensis	neowilforine
Glycyrrhiza uralensis	3,3'-dimethylquercetin
Glycyrrhiza uralensis	dimethyl sebacate
Glycyrrhiza uralensis	narcissin
Glycyrrhiza uralensis	methyl linoleate
Glycyrrhiza uralensis	glycyroside
Glycyrrhiza uralensis	glycyrol
Glycyrrhiza uralensis	18beta-glycyrrhetinic acid
Glycyrrhiza uralensis	methyl 3-o-beta-d-glucopyranosyl polygalacate
Glycyrrhiza uralensis	gloeosteretriol
Glycyrrhiza uralensis	glycyrin
Glycyrrhiza uralensis	licoflavone
Glycyrrhiza uralensis	glycycoumarin
Glycyrrhiza uralensis	lensinine
Glycyrrhiza uralensis	vicianin
Glycyrrhiza uralensis	alpha-trihydroxy coprostanic acid
Glycyrrhiza uralensis	methyl-24-hydroxy-11-deoxoglycyrrhetate
Glycyrrhiza uralensis	methyl 18α-hydroxyglycyrrhetate
Glycyrrhiza uralensis	isoliensinine

Glycyrrhiza uralensis	isolicoflavonol
Glycyrrhiza uralensis	methyl 2-hydroxy-3,4-dimethoxy benzoate
Glycyrrhiza uralensis	licobenzofuran
Glycyrrhiza uralensis	licobichalcone
Glycyrrhiza uralensis	licoisoflavaone
Glycyrrhiza uralensis	licoricesaponine a3
Glycyrrhiza uralensis	licoricesaponine c2
Glycyrrhiza uralensis	licoricesaponine d3
Glycyrrhiza uralensis	licoricesaponine f3
Glycyrrhiza uralensis	licoricesaponine g2
Glycyrrhiza uralensis	licoricesaponine h2
Glycyrrhiza uralensis	glycyrrhetinicacid
Glycyrrhiza uralensis	glycyrrhetol
Glycyrrhiza uralensis	glycyrrhisoflavanone
Glycyrrhiza uralensis	glycyrrhisoflavone
Glycyrrhiza uralensis	lupiwighteone
Glycyrrhiza uralensis	glycyrrhizicacid
Glycyrrhiza uralensis	glyeurysaponin
Glycyrrhiza uralensis	2,5-dihydroxymethyl-3,4-dihydroxypyrrolidine
Glycyrrhiza uralensis	liquoric acid
Glycyrrhiza uralensis	liquiritigenin-7-o-beta-d-(3-o-acetyl)-apiofuranosyl-4'-o-beta-d-glucopyranoside
Glycyrrhiza uralensis	glisoflavanone
Glycyrrhiza uralensis	glyarallin b
Glycyrrhiza uralensis	neomatatabiol
Glycyrrhiza uralensis	2-methyl-1,3,6-trihydroxyanthraquinone
Glycyrrhiza uralensis	3-methyl-6,7,8-trihydropyrrolo[1,2-a]pyrimidin-2-one
Glycyrrhiza uralensis	licochalcone a
Glycyrrhiza uralensis	licocoumarone
Glycyrrhiza uralensis	formononetin
Glycyrrhiza uralensis	3-o-[β-d-glucuronopyranosyl-(1→2)-o-β-d-glucuronopyranosyl]-24-hydroxyglabrolide
Glycyrrhiza uralensis	glyurallin a
Glycyrrhiza uralensis	glyyunnanprosapogenin d
Glycyrrhiza uralensis	glyuranolide
Angelica sinensis	guanosine
Angelica sinensis	retinol
Angelica sinensis	glycyrrhizic acid
Angelica sinensis	campheoside i
Angelica sinensis	crinamine

Angelica sinensis	2',4'-dihydroxyacetophenone
Angelica sinensis	camphene
Angelica sinensis	campherenol
Angelica sinensis	eucalyptin
Angelica sinensis	1-methyl-2-dodecyl-4-(1h)-quinolone
Angelica sinensis	p-cresol
Angelica sinensis	tridecane
Angelica sinensis	cnidilin
Angelica sinensis	vitamin b12
Angelica sinensis	alloocimene
Angelica sinensis	cnidilide
Angelica sinensis	alpha-terpineol
Angelica sinensis	dimethyl camphorate
Angelica sinensis	alpha-chamigrene
Angelica sinensis	phenylacetic acid
Angelica sinensis	linalyl acetate
Angelica sinensis	2,4,5-trimethylbenzaldehyde
Angelica sinensis	vanillin acetate
Angelica sinensis	2,4-dimethylbenzaldehyde
Angelica sinensis	1-dodecene
Angelica sinensis	ligustilide dimer
Angelica sinensis	o-cresol
Angelica sinensis	m-cresol
Angelica sinensis	myristic acid
Angelica sinensis	myristicin
Angelica sinensis	beta-acoradiene
Angelica sinensis	gamma-acoradiene
Angelica sinensis	stigmasteryl ferulate
Angelica sinensis	alpha-acoradiene
Angelica sinensis	dimethyl phthalate
Angelica sinensis	Δ2,4-dihydrophthalicanhydride
Angelica sinensis	dihydropinosylvin
Angelica sinensis	β-bisabolene
Angelica sinensis	dimethyl azelate
Angelica sinensis	1,2-dimethylbenzene
Angelica sinensis	3(s)-3-butyl-4,5-dihydrophthalide
Angelica sinensis	3-carene
Angelica sinensis	3-butylidene-phalide
Angelica sinensis	1-hexadecanol

Angelica sinensis	stigmasterol-β-d-glucoside
Angelica sinensis	3-o-tetradecanoyl-1-cyano-2-methyl-1,2-propene
Angelica sinensis	stigmasterol
Angelica sinensis	2-methyl-dodecane-5-one
Angelica sinensis	suchilactone
Angelica sinensis	succinicacid
Angelica sinensis	sec-butyl isothiocyanate
Angelica sinensis	palmitic acid
Angelica sinensis	anisic acid
Angelica sinensis	lignocericacid
Angelica sinensis	12-o-nicotinoylisolineolone
Angelica sinensis	brefeldin a
Angelica sinensis	choline
Angelica sinensis	uracil
Angelica sinensis	uralene
Angelica sinensis	20-hexadecanoylingenol
Angelica sinensis	z-3',8',3'α,7'α-tetrahydro-6,3',7,7'α-diligustilide-8'-one
Angelica sinensis	hexadecanoicacid
Angelica sinensis	cumaldehyde
Angelica sinensis	beta-elemene
Angelica sinensis	β-chamigrene
Angelica sinensis	1-tridecene
Angelica sinensis	delta-acoradiene
Angelica sinensis	2,4,6-trimethylbenzaldehyde
Angelica sinensis	isoimperatorin
Angelica sinensis	cedrol
Angelica sinensis	2,5-dimethylbenzaldehyde
Angelica sinensis	3,4-dihydroxyallylbenzene 4-o-[alpha-l-rhamnopyranosyl-(1-6)]-beta-d-glucopyranoside
Angelica sinensis	1,8-dimethyl-4-(1-methylenyl)-spiro(4,5)dec-7-ene
Angelica sinensis	(-)-guaia-1(10),11-dien-15-al
Angelica sinensis	naphthalene1
Angelica sinensis	chrysanthemaxanthin
Angelica sinensis	1,1,5-trimethyl-2-formyl-cyclohexa-2,5-diene-4-one
Angelica sinensis	6-undecanone
Angelica sinensis	n-butylidene phthalide
Angelica sinensis	uridine
Angelica sinensis	vitamin b15
Angelica sinensis	phyllanthin

Angelica sinensis	beta-caryophyllene
Angelica sinensis	folinicacid
Angelica sinensis	foliosidine
Angelica sinensis	2-methyl-3-buten-2-ol
Angelica sinensis	phellatin
Angelica sinensis	β-phellandrene
Angelica sinensis	azelaicacid
Angelica sinensis	nonanal
Angelica sinensis	limocitrin-beta-d-glucoside
Angelica sinensis	naphthalene
Angelica sinensis	6-undecanol
Angelica sinensis	n-valerophenone-o-carboxylicacid
Angelica sinensis	heptanal
Angelica sinensis	anglica polysacharide
Angelica sinensis	z-ligustilide
Angelica sinensis	valerosidatum
Angelica sinensis	alpha-pinene
Angelica sinensis	scopoletin
Angelica sinensis	stigmasterol-beta-d-glucoside
Angelica sinensis	beta-myrcene
Angelica sinensis	isocnidilide
Angelica sinensis	vitamin b1
Angelica sinensis	guaiacol
Angelica sinensis	isofernene
Angelica sinensis	dimethyl sulfone
Angelica sinensis	dimethyl sebacate
Angelica sinensis	hexadecanoic acid
Angelica sinensis	decanal
Angelica sinensis	4-octanone
Angelica sinensis	ferulicacid
Angelica sinensis	bicycloelemene
Angelica sinensis	6-o-e-feruloylajugol
Angelica sinensis	beta-bisabolene
Angelica sinensis	sebacicacid
Angelica sinensis	homosenkyunolide h
Angelica sinensis	δ-acoradiene
Angelica sinensis	tetradecane
Angelica sinensis	1-tetradecanol
Angelica sinensis	α-acoradiene

Angelica sinensis	γ-acoradiene
Angelica sinensis	β-acoradiene
Angelica sinensis	homosenkyunolide i
Angelica sinensis	sebiferic acid
Angelica sinensis	scopolin
Angelica sinensis	limonene
Angelica sinensis	ligustilide
Angelica sinensis	24,24-dimethyl-5alpha-cholesta-8-en-3beta-ol
Angelica sinensis	3-methylbutyl-benzene
Angelica sinensis	carvacrol
Angelica sinensis	angelicin
Angelica sinensis	angelicide
Angelica sinensis	butanoic acid
Angelica sinensis	1,2-benzenedicarboxylic acid
Angelica sinensis	vanillin
Angelica sinensis	m-ethylphenol
Angelica sinensis	p-ethylphenol
Angelica sinensis	ethyl-p-methoxycinnamate
Angelica sinensis	4-ethylresorcinol
Angelica sinensis	phthalicanhydride
Angelica sinensis	carvacrol acetate
Angelica sinensis	adeninenucleoside
Angelica sinensis	adenine
Angelica sinensis	hexanoic acid
Angelica sinensis	ethanol
Angelica sinensis	α-cedrene
Angelica sinensis	phenol
Angelica sinensis	decanoic acid
Angelica sinensis	camphoricacid
Angelica sinensis	azelaic acid
Angelica sinensis	anisicacid
Angelica sinensis	butanal
Angelica sinensis	dodecanol
Angelica sinensis	dodecane
Angelica sinensis	isoeugenol
Angelica sinensis	2,3-dicresol
Angelica sinensis	dodecenoic acid
Angelica sinensis	pentylbenzene
Angelica sinensis	dictamnine

Angelica sinensis	dimethyl-beta-propiothetin
Angelica sinensis	succinic acid(high dose)
Angelica sinensis	isococculidine
Realgar	realgar
Realgar	as
Realgar	as2s3
Realgar	as2s2
Pericarpium Zanthoxyli	methyl eugenol
Pericarpium Zanthoxyli	humulene
Pericarpium Zanthoxyli	alpha-pinene
Pericarpium Zanthoxyli	nerylacetate
Pericarpium Zanthoxyli	sabinene
Pericarpium Zanthoxyli	alpha-phellandrene
Pericarpium Zanthoxyli	geranylacetate
Pericarpium Zanthoxyli	alpha-terpineol
Pericarpium Zanthoxyli	beta-pinene
Pericarpium Zanthoxyli	terpinen-4-ol
Pericarpium Zanthoxyli	eugenol
Pericarpium Zanthoxyli	myrcene
Pericarpium Zanthoxyli	geraniol
Pericarpium Zanthoxyli	limonene
Pericarpium Zanthoxyli	trans-caryophyllene
Pericarpium Zanthoxyli	linalool
Pericarpium Zanthoxyli	p-cymene
Pericarpium Zanthoxyli	terpinyl acetate
Pericarpium Zanthoxyli	estragole

Supplementary Table S2 Eight ingredients present in two herbs of the SMBJT formula.

Ingredient	Herb1	Herb2	Targets
ferulic acid	Cimicifuga foetida	Angelica sinensis	MAPK1\FANCE\TPSG1\CSN1S1\ADIPOQ\CYP1A2\PTGS2\VEGFA\AR\CYP1A1
alpha-terpineol	Angelica sinensis	Pericarpium zanthoxyli	
glycyrrhizic acid	Glycyrrhiza uralensis	Angelica sinensis	MMP2\CCL2\GOT2\IL6\REN\IL18\CCL5\JUNB\CXCL10\IL8\NAT1\HSD11B2\ACPP\CYP11B2\CYP3A4\NR3C2\MGEA5\MMP9
dimethyl sebacate	Glycyrrhiza uralensis	Angelica sinensis	
alpha-pinene	Angelica sinensis	Pericarpium zanthoxyli	CYP3A5\IL1B\SAGE1\SPEN\CYP1A1
6-o-e-feruloylajugol	Cimicifuga foetida	Angelica sinensis	
uralene	Glycyrrhiza uralensis	Angelica sinensis	
limonene	Angelica sinensis	Pericarpium zanthoxyli	CCL2

Supplementary Table S3 The 75 targets correlated with more than 5 ingredients.

Target	Ingredient Numbers	Ingredients
IL6	13	butanoic acid; vitamin d; hexadecanoic acid; visnagin; scopolin; liquiritigenin; palmitic acid; ethanol; monoammonium glycyrrhizinate; retinol; glycyrrhizic acid; phenol; glycyrrhizin
TNF	11	uridine; methylglyoxal; choline; guanosine; butanoic acid; isoliquiritigenin; vitamin d; phenol; caffeic acid; licochalcone a; ethanol
VEGFA	10	retinol; ferulic acid; butanoic acid; vitamin d; formononetin; hispidulin; rutin; scopolin; ethanol; scopoletin
IL8	10	humulene; butanoic acid; glycyrrhizic acid; hexadecanoic acid; phenol; tetrahydropalmatine; palmitic acid; ethanol; glycyrrhizin; monoammonium glycyrrhizinate
AKR1B1	9	methylglyoxal; decanal; butanal; isoliquiritigenin; nonanal; heptanal; vitamin b1; phenylacetic acid; hexanoic acid
PTGS2	8	ferulic acid; vitamin d; glycyrrhizic acid; adenine; isoimperatorin; guaiacol; caffeic acid; ethanol
CCL2	8	limonene; glycyrrhizic acid; hexadecanoic acid; licochalcone a; palmitic acid; ethanol; glycyrrhizin; monoammonium glycyrrhizinate
DECR1	8	methylglyoxal; myristic acid; adenine; hexadecanoic acid; phenol; palmitic acid; ethanol; succinic acid(high dose)
SOD2	8	retinol; butanoic acid; guaiacol; hexadecanoic acid; caffeic acid; palmitic acid; ethanol; succinic acid(high dose)
REN	8	choline; vitamin d; glycyrrhizic acid; hexadecanoic acid; palmitic acid; ethanol; glycyrrhizin; monoammonium glycyrrhizinate
ALB	8	retinol; methylglyoxal; decanoic acid; myristic acid; vitamin d; hexadecanoic acid; palmitic acid; ethanol
NFKB1	7	methylglyoxal; butanoic acid; vitamin d; hexadecanoic acid; palmitic acid; vanillin; ethanol
GCG	7	choline; myristic acid; butanoic acid; hexadecanoic acid; palmitic acid; hexanoic acid; ethanol
OXT	7	choline; butanoic acid; bicuculline; hexadecanoic acid; palmitic acid; hexanoic acid; ethanol
MMP2	7	butanoic acid; vitamin d; glycyrrhizic acid; phenol; ethanol; glycyrrhizin; monoammonium glycyrrhizinate
MMP9	7	retinol; glycyrrhizic acid; phenol; vanillin; ethanol; glycyrrhizin; monoammonium glycyrrhizinate
CAT	7	retinol; guaiacol; phenol; lignocericacid; ethanol; scopoletin; succinic acid(high dose)
CCK	7	guanosine; butanoic acid; vitamin d; hexadecanoic acid; palmitic acid; hexanoic acid; ethanol
CYP1B1	7	retinol; naphthalene; formononetin; isoimperatorin; rutin; umbelliferone; isoquercitrin
KNG1	7	guanosine; butanoic acid; glycyrrhizic acid; hexadecanoic acid; palmitic acid; hexanoic acid; ethanol
BCL2	7	butanoic acid; hispidulin; tridecane; hexadecanoic acid; stigmasterol; palmitic acid; ethanol
NGF	7	retinol; choline; guanosine; butanoic acid; bicuculline; vitamin d; ethanol
CYP3A4	7	vitamin d; glycyrrhizic acid; umbelliferone; liquiritigenin; ethanol; glycyrrhizin; monoammonium glycyrrhizinate
CASP3	7	retinol; 尾-sitosterol; butanoic acid; isoliquiritigenin; hexadecanoic acid;

		palmitic acid; ethanol
HSD11B2	6	glycyrrhetinicacid; 18beta-glycyrrhetinic acid; glycyrrhizic acid; glycyrrhetinic acid; glycyrrhizin; monoammonium glycyrrhizinate
GPR17	6	butanoic acid; glycyrrhizic acid; uracil; hexadecanoic acid; palmitic acid; hexanoic acid
FOS	6	choline; butanoic acid; bicuculline; hexadecanoic acid; palmitic acid; ethanol
BDNF	6	retinol; choline; bicuculline; vitamin d; caffeic acid; ethanol
GOT2	6	retinol; glycyrrhizic acid; hexadecanoic acid; palmitic acid; glycyrrhizin; monoammonium glycyrrhizinate
PRKAA2	6	butanoic acid; adenine; hispidulin; hexadecanoic acid; palmitic acid; ethanol
ICAM1	6	glycyrrhetinicacid; 18beta-glycyrrhetinic acid; butanoic acid; glycyrrhizic acid; phenol; glycyrrhetinic acid
GNRH1	6	butanoic acid; bicuculline; hexadecanoic acid; palmitic acid; hexanoic acid; ethanol
NR3C2	6	glycyrrhetinicacid; 18beta-glycyrrhetinic acid; glycyrrhizic acid; glycyrrhetinic acid; glycyrrhizin; monoammonium glycyrrhizinate
CASR	6	butanoic acid; vitamin d; glycyrrhizic acid; hexadecanoic acid; palmitic acid; hexanoic acid
PLA2G1B	6	methylglyoxal; anisic acid; choline; hexadecanoic acid; palmitic acid; ethanol
GJA1	6	glycyrrhetinicacid; 18beta-glycyrrhetinic acid; 18alpha-glycyrrhetinic acid; brefeldin a; glycyrrhetinic acid; ethanol
CYP1A1	6	retinol; ferulic acid; formononetin; alpha-pinene; ethanol; isoquercitrin
GSR	6	retinol; methylglyoxal; vitamin b1; guaiacol; rutin; ethanol
F2R	6	guanosine; butanoic acid; brefeldin a; hexadecanoic acid; palmitic acid; hexanoic acid
P2RY2	6	uridine; butanoic acid; adenine; hexadecanoic acid; palmitic acid; hexanoic acid
PNP	6	uridine; guanosine; glycyrrhizic acid; adenine; uracil; ethanol
EDN1	6	butanoic acid; hexadecanoic acid; phenol; palmitic acid; hexanoic acid; ethanol
MGEA5	6	glycyrrhetinicacid; 18beta-glycyrrhetinic acid; glycyrrhizic acid; glycyrrhetinic acid; glycyrrhizin; monoammonium glycyrrhizinate
BAX	6	decanoic acid; butanoic acid; hexadecanoic acid; palmitic acid; ethanol; corylifolinin
AKT1	5	guanosine; vitamin d; hexadecanoic acid; palmitic acid; ethanol
FPR2	5	butanoic acid; glycyrrhizic acid; hexadecanoic acid; palmitic acid; hexanoic acid
FASN	5	vitamin d; hexadecanoic acid; caffeic acid; palmitic acid; ethanol
PPARG	5	glycyrrhizic acid; hexadecanoic acid; palmitic acid; ethanol; scopoletin
ADIPOQ	5	ferulic acid; glycyrrhizic acid; hexadecanoic acid; palmitic acid; ethanol
SRC	5	glycyrrhetinicacid; myristic acid; 18beta-glycyrrhetinic acid; guanosine; glycyrrhetinic acid
LPAR1	5	butanoic acid; glycyrrhizic acid; hexadecanoic acid; palmitic acid; hexanoic acid
LPAR2	5	butanoic acid; glycyrrhizic acid; hexadecanoic acid; palmitic acid; hexanoic acid
LPAR3	5	butanoic acid; glycyrrhizic acid; hexadecanoic acid; palmitic acid; hexanoic acid
TH	5	retinol; choline; butanoic acid; vitamin d; ethanol
GNRHR	5	myristic acid; butanoic acid; hexadecanoic acid; palmitic acid; hexanoic acid
IL18	5	butanoic acid; glycyrrhizic acid; ethanol; glycyrrhizin; monoammonium

		glycyrrhizinate
RXRA	5	retinol; vitamin d; hexadecanoic acid; palmitic acid; ethanol
TAC1	5	butanoic acid; bicuculline; hexadecanoic acid; palmitic acid; hexanoic acid
GNG2	5	butanoic acid; glycyrrhizic acid; hexadecanoic acid; palmitic acid; hexanoic acid
ACOT4	5	decanoic acid; myristic acid; hexadecanoic acid; palmitic acid; succinic acid(high dose)
ANXA1	5	butanoic acid; glycyrrhizic acid; hexadecanoic acid; palmitic acid; hexanoic acid
CHRM1	5	butanoic acid; hexadecanoic acid; palmitic acid; hexanoic acid; ethanol
CHRM5	5	butanoic acid; hexadecanoic acid; rutin; palmitic acid; hexanoic acid
TRH	5	butanoic acid; hexadecanoic acid; palmitic acid; hexanoic acid; ethanol
P2RY6	5	uridine; butanoic acid; hexadecanoic acid; palmitic acid; hexanoic acid
P2RY1	5	butanoic acid; adenine; hexadecanoic acid; palmitic acid; hexanoic acid
LEP	5	retinol; butanoic acid; hexadecanoic acid; palmitic acid; ethanol
APP	5	butanoic acid; glycyrrhizic acid; hexadecanoic acid; palmitic acid; hexanoic acid
EDNRB	5	butanoic acid; vitamin d; hexadecanoic acid; palmitic acid; hexanoic acid
TYR	5	p-cresol; azelaic acid; azelaicacid; phenol; caffeic acid
PRL	5	choline; butanoic acid; bicuculline; vitamin d; ethanol
TLR4	5	myristic acid; vitamin d; hexadecanoic acid; palmitic acid; ethanol
NTS	5	butanoic acid; bicuculline; hexadecanoic acid; palmitic acid; hexanoic acid
BDKRB2	5	butanoic acid; glycyrrhizic acid; hexadecanoic acid; palmitic acid; hexanoic acid
BDKRB1	5	butanoic acid; glycyrrhizic acid; hexadecanoic acid; palmitic acid; hexanoic acid

Supplementary Table S4 The top biological processes enriched in SMBJT targets.

**Term**	**Count**	**%**	**P-Value**	**Benjamini**
signal transduction	341	29.1	2.10E-17	1.70E-15
cell surface receptor linked signal transduction	286	24.4	6.80E-31	4.20E-28
positive regulation of cellular process	262	22.4	1.90E-22	4.40E-20
organ development	223	19	1.40E-13	6.80E-12
negative regulation of cellular process	196	16.7	9.10E-09	2.30E-07
intracellular signaling cascade	192	16.4	1.20E-19	1.60E-17
regulation of cell communication	150	12.8	5.20E-13	2.30E-11
oxoacid metabolic process	149	12.7	1.10E-41	2.00E-38
regulation of cell proliferation	147	12.5	4.50E-23	1.20E-20
chemical homeostasis	140	11.9	3.60E-40	3.30E-37
regulation of cell death	118	10.1	2.30E-10	6.80E-09
cellular lipid metabolic process	117	10	8.30E-25	3.00E-22
regulation of programmed cell death	117	10	3.60E-10	1.00E-08
positive regulation of metabolic process	117	10	0.00000048	0.0000077

Supplementary Table S5 A total of 235 target genes present in the 28 functional modules.

**Modules**	**Proteins**
M1.1	KIAA0101
M1.2	PRTFDC1,P2RY12,EGF,THBS1,PPBP,CA2,SDPR,SELP,MMD,ALOX12,CXCL5,TBXA2R,SDPR,MMD
M1.3	MYC,CD79A
M1.4	BTG3,TFRC,PDE4DETNK1,JUND,MECP2
M1.5	SLC27A1,CD14,LILRB2,KYNU,TLR2,GM2A,CD86,IMPDH1,CTSB,ASGR1,CTSS,VDR,MYD88,ACPP,TNFRSF1B
M1.6	SMG1,PPP3CA
M1.7	RPL13A,TXNIP,RPS3,EEF2,B2M,RPL22
M1.8	NAT1
M2.1	APOBEC3F,CCL5,GNLY,FYN
M2.2	MMP9,CEACAM1,TCN1,CAMP,LCN2,ARG1,HBG2
M2.3	BCL2L1,HBA1,CHPT1,DRD2,SLC14A,HBG1,HBG2,EPB49,CARM1,TBXA2R,HAGH,CA1
M2.4	ATP5O,AKR1B1,RPL22,APRT,EEF2,RPL13A,C19orf2
M2.5	CSN2,CA1,THBS1,MMP14,CD80,CYP2C9,NTRK2,CHST4,HRH4,AGTR1,GAL,RARB,MTAP,GSTA1,ERBB3,CD40,HOXB5,CTSE,CYP2A6,P2RY4
M2.6	RBP7,INSR,SDCBP,FPR1,TNFRSF1A,CDA,CTSB,SLC19A1,MITF,ALDH2,KYNU,PTEN,CYP1B1,FBP1,ALOX5,BRI3,PTAFR,RXRA
M2.7	PRKAR2A,SLC22A3,SFTPB
M2.8	PKIA,PRKCA,LDHB,NMT2,CAMK4,CCR7,AQP3,FLT3LG
M2.9	MAPK1, RAB5A,FAS,ITGA4
M2.10	CD36,CD86,LILRB
M2.11	CDK6,PRKCI,ITPKB,STK17B,PIK3R1,KRAS
M3.1	AGRN,SERPING1,STAT1,EIF2AK2,TNFSF10,NT5C3,CXCL10
M3.2	IL8,ICAM1,CD44,PLAUR,IL1A,CXCL16,MCL1,RARA,BCL3,BCL6,BCL2A1
M3.3	IL18, ALOX5, ANPEP, HMOX1, PPT1,CTSB,CTSS, ASAH1,CAST
M3.4	PPP1R12A, PTPRC,PIK3CA
M3.5	HBA1,HBB
M3.6	GFM1,ATP6V1C,ATP6V1D
M3.7	PHPT1,ACO2,ACSL5,RABEP1
M3.8	METTL3,NR2C2,OGG1,TBP,MBD4,C19orf2,NMT2,TFB1M,LARS
M3.9	PRKDC, PRKCI,PTPRC, MTMR2
